# A Coupled Thermochemical Model for Predicting Fire-Induced Thermal Responses and Decomposition Behavior

**DOI:** 10.3390/polym17070939

**Published:** 2025-03-30

**Authors:** Bin Wu, Wenguo Weng, Tai Zeng, Zuxi Xia, Zhengliang Su, Fei Xie

**Affiliations:** 1 The Second Research Institute of CAAC, Chengdu 610041, China; wubin@fccc.org.cn (B.W.); xiazuxi@fccc.org.cn (Z.X.); suzhengliang@fccc.org.cn (Z.S.); xiefei@fccc.org.cn (F.X.); 2 Key Laboratory of Aviation Fuel Airworthiness and Green Development of Civil Aviation, Chengdu 610041, China; 3 School of Safety Science, Institute of Public Safety Research, Tsinghua University, Beijing 100084, China; wgweng@tsinghua.edu.cn

**Keywords:** composite materials, mathematical model, heat transfer, finite element

## Abstract

Composite materials are increasingly used in aerospace applications due to their high strength-to-weight ratio, but their fire safety remains a critical concern. This study develops a coupled thermochemical model to predict the thermal response and decomposition behavior of composite materials under high-temperature fire conditions. The framework integrates heat transfer, resin pyrolysis kinetics, and gas generation dynamics, employing the Rule of Mixtures to dynamically update temperature-dependent thermophysical properties (thermal conductivity, specific heat capacity, and density). Decomposition kinetics are governed by an n-th-order Arrhenius equation, explicitly resolving the gas convection effects on energy transport. The governing equations are solved numerically using a hybrid explicit/implicit finite element scheme, ensuring stability under severe thermal gradients. Experimental validation compliant with the 14 CFR Part 25 and ISO 2685 standards demonstrates high predictive accuracy. The model successfully captures key phenomena, including the char layer insulation effects, transient heat flux attenuation, and decomposition-induced property transition. This work establishes a computational foundation for optimizing fire-resistant composites in aerospace applications, addressing critical gaps in the existing models through coupled multiphysics representation.

## 1. Introduction

Composite materials have become indispensable in various industries, particularly in aerospace, automotive, and construction applications, owing to their exceptional strength-to-weight ratio, superior corrosion resistance, and remarkable design flexibility. In modern civil aircraft design, composite materials are progressively replacing the traditional metallic components in critical structural elements, including wings, empennage assemblies, and fuselage skins, primarily due to their enhanced mechanical properties and significant weight reduction potential [1]. Nevertheless, fire safety considerations remain a paramount concern, as the fire resistance characteristics of fuselage composites critically influence both the structural integrity of the airframe and passenger survival probability during fire incidents. Unlike the conventional metallic materials, polymer-based composites demonstrate complex pyrolysis and combustion behaviors, characterized by rapid thermal degradation, substantial volatile gas emission, and the formation of brittle carbonaceous residues. These phenomena not only lead to severe mechanical performance degradation, but also pose significant challenges in developing materials capable of withstanding extreme thermal environments. Addressing these challenges necessitates a comprehensive understanding of the heat transfer mechanisms and the thermal decomposition processes in composites under fire conditions. Consequently, research focused on enhancing composite fire resistance and mitigating fire propagation has become a critical area of study in advancing civil aviation safety standards.

Extensive research efforts have significantly advanced the understanding of composite material behavior under high-temperature conditions, establishing a robust theoretical foundation for optimizing composite design and developing rigorous fire safety standards. Shi et al. [2] conducted a comprehensive review of one-dimensional models for combustible materials exposed to high temperatures, systematically analyzing the key physical and chemical processes, including heat transfer mechanisms, pyrolysis kinetics, gas volatilization, volumetric changes, moisture evaporation, internal pressure development, permeability variations, and mechanical property degradation. Progelhof [3] critically evaluated both theoretical and empirical methods for predicting the thermal conductivity of composites, highlighting the inherent limitations of the existing models and emphasizing the lack of a universal predictive framework applicable across diverse composite systems. Henderson et al. [4,5] made significant contributions by developing a transient thermal model capable of simulating the complex decomposition and expansion behaviors of composites at elevated temperatures. Building upon this foundation, Gibson [6,7,8] established an advanced modeling framework for analyzing the thermal response of composite laminates in fire conditions, successfully integrating laminate structural analysis with thermal ablation modeling to predict both the temperature distributions and resin content variations. Gibson further extended this work by proposing an innovative “double-layer” model for assessing the structural response of laminates under fire exposure conditions. Schuhler et al. [9] conducted pioneering experimental simulations of aircraft engine fire scenarios using controlled kerosene burner tests, providing valuable insights into the ignition behaviors of various composite materials and demonstrating significant material-dependent variations in fire performance and thermal protection characteristics.

In the context of fire-exposed wooden structures, Kansa [10] developed an advanced one-dimensional mathematical model for char pyrolysis that effectively captures the complex interactions of volatile flow within porous, permeable materials. This sophisticated model incorporates temperature-dependent thermal properties, employs a pyrolysis reaction framework based on the Arrhenius equation, and utilizes Darcy’s law to characterize anisotropic transport phenomena during wood heating. The model’s comprehensive approach successfully addresses the intricate coupling of heat and mass transfer mechanisms in lignocellulosic materials. Similarly, Fredlund et al. [11] proposed a robust analytical model to describe the coupled heat and mass transfer behaviors of wood under fire conditions. Their model considers transient temperature and moisture content distributions in both uncharred and charred regions, while also incorporating the dynamic growth and oxidation kinetics of the char layer. Through the integration of these critical factors, the model significantly enhances the predictive accuracy of thermal degradation processes in wooden structures during fire exposure.

Zhang et al. [12] developed an innovative three-dimensional thermodynamic model capable of predicting the thermal response of polymer composites across a broad temperature spectrum. This comprehensive model incorporates three critical phenomena, viscoelastic behavior, thermal decomposition kinetics, and thermal expansion effects, with governing equations that explicitly account for the decomposition reactions and the gas storage dynamics. In a complementary study, Chang [13] conducted an in-depth investigation of the thermal effects on polymer composites under fire conditions, with a particular emphasis on the synergistic roles of moisture content and heat transfer in material degradation processes. Mouritz et al. [14,15,16,17,18] made significant contributions through their systematic investigation of fire behavior in polymer composites, establishing a comprehensive framework that encompasses the thermal degradation mechanisms, the combustion characteristics, and the fire resistance properties.

Bai et al. [19] conducted a comprehensive investigation of the thermal properties of fiber-reinforced plastic (FRP) composites, developing a sophisticated resin decomposition model based on chemical kinetics principles that significantly enhanced the understanding of composite behavior under elevated temperatures. Ramroth et al. [20] constructed an advanced thermal model specifically for FRP composites exposed to fire conditions, providing valuable insights into their thermal response characteristics. Looyeh [21,22] performed detailed finite element analyses on glass-reinforced plastic (GRP) systems, examining the combined effects of environmental humidity and temperature-dependent thermal properties on the nonlinear boundary conditions of unexposed surfaces, thereby improving the accuracy of fire performance predictions. Zhang et al. [23] conducted the systematic evaluation of the protective mechanisms of fire-retardant layers in glass fiber-reinforced polymer (GFRP) composites, developing a comprehensive finite element heat transfer model to assess the thermal response under fire conditions, with a particular emphasis on the loading conditions, layer thickness optimization, and the material selection criteria. Zhang et al. [24] further advanced the field by introducing a two-dimensional thermodynamic model specifically designed to capture the complex fire response of GFRP composites. Dodds et al. [25] performed extensive experimental investigations of the thermal response of GFRP under severe fire conditions, identifying the critical performance factors, such as low thermal conductivity and matrix decomposition kinetics. Their work was subsequently extended to develop predictive capabilities for temperature distributions, thermal stresses, and fire risks in complex structural components. Luo et al. [26,27,28] made significant contributions by proposing a novel thermo-mechanical damage model for glass/phenolic composites in high-temperature environments, employing advanced homogenization techniques and elasticity equations to model gas transport phenomena and mechanical interactions, substantially advancing the understanding of composite damage mechanisms under extreme thermal conditions.

James [29] conducted a fundamental investigation into the transverse thermal conductivity of fiber-reinforced composites, providing critical insights into their anisotropic heat transfer characteristics. Kandare et al. [30] performed the extensive validation of a thermodynamic model for fiber-reinforced composites under high-temperature conditions using experimental data, while systematically exploring various fire-retardant systems to enhance the fire resistance properties. Miano [31] introduced an innovative modeling approach incorporating apparent thermal diffusivity (ATD), which significantly reduced computational complexity, while maintaining the accurate prediction of decomposition behavior under high heat flux conditions. Tranchard [32,33] conducted comprehensive studies of the thermal properties of carbon fiber-reinforced polymer (CFRP) laminates using advanced characterization techniques, including Thermogravimetric Analysis (TGA), Simultaneous Thermal Analysis (STA), Laser Flash Analysis (LFA), and Fourier Transform Infrared Spectroscopy (FTIR). Furthermore, he developed a sophisticated three-dimensional thermochemical model capable of predicting mass loss kinetics and pyrolysis behaviors. Despite these significant advancements, their findings underscored persistent challenges in resolving temperature distribution uncertainties, primarily due to the inherent variability in flame heat flux conditions.

Lattimer [34] developed a comprehensive model to characterize heat and mass transfer phenomena in CFRP under fire conditions, incorporating critical factors such as thermochemical expansion, gas storage dynamics, and extensive experimental validation to ensure predictive accuracy. Chippendale et al. [35] conducted a detailed investigation of the thermal decomposition and damage mechanisms in CFRP under high heat flux conditions, revealing that internal gas pressure plays a crucial role in driving crack formation and delamination processes, thereby emphasizing the significance of gas transport mechanisms in structural damage progression. Summers et al. [36,37] introduced an advanced thermal–structural model for fiber-reinforced polymer laminates subjected to one-sided fire heating, providing valuable insights into temperature distribution patterns within sandwich composite structures. Feih et al. [38,39,40] made significant advancements in thermodynamic modeling to assess the residual compressive strength and failure time of sandwich composites under fire conditions, incorporating critical considerations such as heat conduction mechanisms, matrix decomposition kinetics, and gas exhaust processes. Gu et al. [41,42,43] systematically studied the effects of transverse thermal gradients on the wrinkling load capacity of polymer-based sandwich panels, highlighting the critical influence of geometric dimensions and material degradation characteristics. Upasiri et al. [44] employed sophisticated finite element analysis techniques to evaluate the fire resistance of sandwich composites, establishing a robust computational framework for assessing composite behavior in high-temperature environments.

In summary, substantial progress has been achieved in understanding the high-temperature behavior of composite materials through the development of sophisticated heat transfer and thermal decomposition models. These models, spanning from one-dimensional to three-dimensional formulations, systematically integrate critical factors, including material degradation kinetics, gas generation mechanisms, and thermal decomposition reactions, to analyze the thermal response characteristics, the combustion processes, and thermophysical property variations under fire conditions. Extensive experimental validation has significantly enhanced their reliability and predictive capabilities. However, the existing models exhibit several notable limitations. Most formulations lack a comprehensive description of the phase transition mechanisms and fail to account for the dynamic effects of phase transitions on the thermophysical properties. The treatment of gas generation dynamics and transport phenomena is often oversimplified, potentially leading to the underestimation of gas pressure effects on the structural performance. Furthermore, thermal–mechanical coupling remains insufficiently explored, with limited consideration given to the degradation of mechanical properties at elevated temperatures and their interaction with heat transfer processes, thereby restricting the models’ applicability in complex fire scenarios.

To address these critical limitations, this study develops an advanced integrated mathematical model that incorporates coupled heat transfer mechanisms, thermal decomposition reactions, and gas generation processes in composite materials under high-temperature conditions. The model dynamically calculates the variations in thermophysical properties using the Rule of Mixtures approach, effectively overcoming the previous deficiencies in phase transition descriptions. It employs the Arrhenius equation for kinetic modeling of matrix decomposition reactions, quantitatively describes the gas generation rates, and accurately captures the coupling effects between gas dynamics and heat transfer, thereby addressing the oversimplifications prevalent in prior work. Additionally, the model supports multiple boundary condition configurations, significantly enhancing its relevance to practical engineering applications. The experimental methodology adheres to the 14 CFR Part 25 [45] and ISO 2685 [46] testing standards, utilizing high-precision thermocouples and heat flux meters to validate predicted temperature distributions and heat flux profiles. The experimental results demonstrate excellent agreement with the numerical simulations, with a maximum deviation of less than 5%, confirming the reliability and robustness of the proposed model for engineering applications.

## 2. Materials and Methods

### 2.1. Basic Assumptions

When composite materials are exposed to fire conditions, they undergo a series of complex physical and chemical transformations as the temperature increases, which can be systematically categorized into distinct phases. The process begins with radiative heat flow, where thermal radiation from the fire source penetrates the front surface of the composite laminate, initiating the heating process. During the transient heat conduction phase, the surface temperature remains relatively low (typically below 200–300 °C, depending on material composition, the processing parameters, and the heating rate), with no significant chemical reactions occurring, although the surface temperature exhibits rapid temporal variations due to transient heat transfer effects. As the surface temperature reaches critical levels sufficient to initiate thermal degradation, the material enters the pyrolysis phase, undergoing thermal decomposition that results in char layer formation and the release of gaseous decomposition products. During this phase, the chemical reaction zone progressively propagates inward from the heated surface, while thermochemical processes, including expansion or contraction, occur in response to elevated temperatures. The accumulation of decomposition gases within the material matrix leads to internal pressurization, and the temperature gradient across the material gradually diminishes, maintaining relatively low temperatures in the unreacted solid regions. For the gas phase, several key assumptions are made: decomposition gases disperse completely without accumulation in the solid phase; thermal expansion effects are excluded from the current model formulation; thermal equilibrium is maintained between decomposition gases and the solid phase throughout the process; resin sublimation occurs without intermediate compound formation; the thermal and transport properties of the laminate are treated as constant during decomposition (though temperature-dependent in nature); no solid-phase material enters the system’s control volume; solid-to-gas transformation is modeled as a continuous mass loss process; decomposition kinetics are described using a first-order Arrhenius equation; and material density approaches zero upon complete decomposition.

### 2.2. Energy Conservation

To simulate the thermal degradation of composite materials under fire exposure, the proposed model employs a one-dimensional framework representing an infinite flat composite laminate of finite thickness *L*, with heat transfer restricted to the through-thickness direction (*x*-axis). The heating scenario is governed by either a uniform incident heat flux or a prescribed surface temperature applied to the exposed face (*x* = 0).

The material system is partitioned into two distinct phases:

(1) Solid Phase: Comprising the undamaged matrix, reinforcing fibers, and char residues formed during decomposition ($\rho_{s}=\rho_{fiber}+\rho_{char}$).
(2) Gas Phase: Consisting of pyrolysis volatiles, entrapped air, water vapor, and soot particles ($\rho_{g}=\sum_{j}Y_{j}\rho_{g}$, where *Y_j_* is the mass fraction of gas species *j*).

Structural deformations are excluded based on the focus on early-stage pyrolysis-dominated degradation. Radiation absorption by soot-laden pyrolysis gases is modeled via the equivalent diffusion approximation, replacing the radiative transfer equation (RTE) with a computationally tractable diffusion approximation.

Within a differential control volume from (x to x + Δx), the energy balance accounts for heat conduction, radiation, gas-phase enthalpy transport, and any internal heat sources or sinks, yielding the following:

(1) $$\frac{\partial U}{\partial t}=\frac{\partial }{\partial x}\left(\lambda_{eff}\frac{\partial T}{\partial x}\right)+\frac{\partial }{\partial x}\left(\frac{16\sigma T^{3}}{3\beta_{R}}\frac{\partial T}{\partial x}\right)-\frac{\partial }{\partial x}\left({{\dot{m}}_{g}h}_{g}\right)+\tilde{s}$$

where U=ρh is volumetric internal energy density (J⋅m^−3^), $\rho =\rho_{s}+\rho_{g}$ is total density. λ_eff_ is the effective thermal conductivity (W⋅m^−1^⋅K^−1^), dependent on porosity *Φ* and temperature *T. β_R_* is the total extinction coefficient for radiation (m^−1^), accounting for absorption (*β_a_*_,*j*_) and scattering (*β_s_*_,*j*_) by gas species and soot, $\tilde{s}$ is the volumetric heat source (W⋅m^−3^) dominated by endothermic pyrolysis reactions.

*q* is the volumetric heat source (W·m^−3^), representing the heat absorbed by endothermic pyrolysis reactions. Total heat flux *q* includes contributions from Fourier heat conduction and radiative diffusion:

(2) $$q=-\left(\lambda_{eff}+\frac{16\sigma T^{3}}{3\beta_{R}}\right)\frac{\partial T}{\partial x},\;\;\beta_{R}=\sum_{j}Y_{j}\left(\beta_{a,j}+\beta_{s,j}\right)+\beta_{soot}Y_{soot}$$

where *β*_soot_ is the soot absorption coefficient (m^−1^).

*Y*_soot_ is the soot mass fraction governed by Arrhenius kinetics:

(3) $$\frac{\partial Y_{soot}}{\partial t}=A_{soot}exp\left(-\frac{E_{soot}}{RT}\right)\cdot \left(1-\Phi \right),\;Y_{soot}\left(x,0\right)=0,\;{\;\;\;\;\left.\frac{\partial Y_{soot}}{\partial x}\right|}_{x=0,L}=0$$

where *A*_soot_ and *E*_soot_ are pre-exponential factor (s^−1^) and activation energy (J⋅mol^−1^) for soot formation. *Φ* is porosity, $1-\rho /\rho_{0}$, which presents soot generation in highly porous regions.

Effective thermal conductivity *λ*_eff_ is modeled as the volume-weighted average of the solid and gas phases:

(4) $$\lambda_{eff}=\lambda_{solid}\left(1-\Phi \right)+\lambda_{gas}\Phi$$

where λ_solid_ is solid-phase conductivity (W⋅m^−1^ K^−1^), summing the contributions from the fibers and the char. λ_gas_ is gas-phase conductivity (W⋅m^−1^ K^−1^), calculated as λ_gas_ = ∑_j_*Y*_j_λ_j_(*T*).

Pyrolysis gas mass flux ${\dot{m}}_{g}$ follows a modified Darcy–Forchheimer relation:

(5) $${\dot{m}}_{g}=\frac{\lambda \left(\Phi \right)}{\mu }\frac{\partial P}{\partial x}{\left(1+\left[\frac{\rho_{g}\left|m_{g}\right|}{\mu \beta k\left(\Phi \right)}\right]\right)}^{-1},\;\;\lambda \left(\Phi \right)=\lambda_{0}{\left(\frac{\Phi }{\Phi_{0}}\right)}^{3}$$

where *k*(*Φ*) is permeability (m^2^), scaling with *Φ*^3^ to reflect pore connectivity effects.

Steady-state mass conservation links gas flux to the pyrolysis rate:

(6) $$\dot{G}=\frac{\partial {\dot{m}}_{g}}{\partial x}=-\frac{\partial \rho_{r}}{\partial t}$$

The volumetric heat source $\tilde{s}$ quantifies energy consumption during multi-step pyrolysis:

(7) $$\tilde{s}=-\sum_{i=1}^{N}Qi\frac{\partial \rho_{r,i}}{\partial t}$$

where *Q_i_* is the heat of decomposition (J⋅kg^−1^) for reaction *i*.

The reaction rates follow a char-inhibited Arrhenius model:

(8) $$\frac{\partial \rho_{r,i}}{\partial t}=A_{i}e^{-\frac{E_{i}}{RT}}\rho_{r,i}{\left(1-\frac{\rho_{r,i}}{\rho_{r,i}^{0}}\right)}^{n_{i}}\cdot \xi \left(a\right),\;\;\;\xi \left(a\right)=1-a_{char},\;\;\;a_{char}=\frac{\rho_{char}}{\rho_{solid}}$$

Surface emissivity *ϵ* evolves with char formation:

(9) $$\epsilon =\epsilon_{virgin}\left(1-a_{char}\right)+\epsilon_{char}a_{char}$$

This study establishes a one-dimensional thermal response model for composite materials under fire exposure through the systematic integration of energy conservation (Equation (1)), mass transport (Equations (5) and (6)), and pyrolysis kinetics (Equations (3), (7) and (8)). The energy balance framework combines conductive heat transfer modulated by porosity-dependent effective conductivity *k*_eff_ (Equation (4)), radiation diffusion approximated via a temperature-dependent extinction coefficient *β_R_* (Equation (2)), and convective enthalpy transport by pyrolysis gases ${\dot{hm}}_{g}$. Mass conservation explicitly links the gas generation rates *G* (Equation (6)) to Arrhenius-driven solid decomposition (Equation (8)), while temperature evolution (Equation (9)) incorporates dynamic surface emissivity changes during char formation. The model uniquely resolves heat source coupling between endothermic resin pyrolysis and exothermic char formation (Equation (7)), with soot–radiation feedback (Equation (3)) and permeability–porosity scaling (Equation (5)) enhancing physical fidelity.

### 2.3. Solid-Phase Composition

If the experimental data for the properties of the original composite material are unavailable, the material parameters can be approximated using the properties of the matrix and the reinforcing fibers. In this study, the Rule of Mixtures is employed to estimate the density and thermal properties of the solid phase for the original composite material.

(10) $$\rho_{v}=V_{f}\rho_{vf}+(1-V_{f})\rho_{vr}$$

(11) $$\lambda_{v}={\lambda_{vf}V}_{f}+\lambda_{vr}\left(1-V_{f}\right)$$

(12) $$c_{pv}={c_{pvf}V}_{f}+c_{pvr}\left(1-V_{f}\right)$$

The volume fraction of the reinforcing fibers in the original composite material is denoted by $V_{f}$. The specific heat capacity of the original composite material is represented by $c_{pv}$ (J kg^−1^ K^−1^), with $c_{pvf}$ and $c_{pvr}$ denoting the specific heat capacities of the reinforcing fibers and the matrix, respectively (J kg^−1^ K^−1^). The thermal conductivity of the original composite material is denoted by $k_{v}$ (W m^−1^ K^−1^), while $\lambda_{vf}$ and $\lambda_{vr}$ represent the thermal conductivities of the reinforcing fibers and the matrix, respectively (W m^−1^ K^−1^).

Assuming the resin matrix decomposes into gas and char residue, the solid phase evolves as a mixture of fibers, residual char, and dynamically generated pores. The decomposition progress is quantified by mass fraction *F*:

(13) $$F=\frac{\rho_{r}-\rho_{ch}}{\rho_{vr}-\rho_{ch}}$$

The mass density of the matrix is expressed as follows:

(14) $$\rho_{r}=F\rho_{vr}+\left(1-F\right)\rho_{ch}$$

The density and thermal properties of the composite material during matrix decomposition can be estimated using the Rule of Mixtures as follows:

(15) $$\rho =V_{f}\rho_{vf}+(1-V_{f})\rho_{r}$$

The thermal conductivity integrates porosity–radiation coupling and replaces the original series mixing rule with a unified formulation:

(16) $$\lambda =\left(1-\Phi \right)\left[\left(V_{f}\lambda_{vf}+F(1-V_{f})\lambda_{vf}\right)\right]+\Phi \left(\lambda_{air}+\frac{16\sigma T^{3}}{3\beta_{R}}\right)$$

Post-decomposition char conductivity combines the fiber skeleton, the gas pores, and radiation:

(17) $$\lambda_{char}=\left(1-\Phi_{char}\right)\left[\left(V_{f}\lambda_{vf}+(1-V_{f})\lambda_{vf}\right)\lambda_{char}^{0}\right]+\Phi_{char}\lambda_{gas}+\frac{16\sigma T^{3}}{3\beta_{R}}$$

A quasi-steady three-stage Arrhenius model formulation:

(18) $$\frac{\partial \rho_{r}}{\partial t}=-\sum_{i=1}^{3}A_{i}(\rho_{vr,i}-\rho_{ch,i})F_{i}^{n_{i}}exp\left(-\frac{E_{a}}{RT}\right)$$

Permeability follows a power law relationship with porosity:

(19) $$k=k_{0}\Phi^{2.5}$$

This section systematically derives the evolution of the density and thermal properties of composite materials during matrix decomposition under fire conditions through Equations (10)–(19), aiming to comprehensively analyze their thermal response behavior. The axial thermal conductivity of the original composite is first modeled via a parallel mixing rule (Equation (11)), which aligns with the unidirectional alignment of reinforcing fibers, providing higher accuracy than the traditional series models. During resin decomposition, porosity *Φ* dynamically couples with radiation heat transfer through a unified thermal conductivity formulation (Equation (16)). For the char layer, thermal conductivity (Equation (17)) integrates the residual fibers, gas-filled pores, and radiation effects under the Rosseland approximation, validated for scenarios where material thickness exceeds the photon mean free paths. The decomposition kinetics are upgraded to a quasi-steady three-stage model (Equation (18)). Gas transport is governed by a porosity-dependent permeability law (Equation (19)).

### 2.4. Initial Conditions

The initial conditions for the system are specified as follows:

(20) $${\rho (0)=\rho }_{v}$$

(21) $${T(0)=T}_{ini}$$

where $\rho_{v}$ is the mass density of the original composite material (kg m^−3^), and $T_{ini}$ is the initial temperature (K).

### 2.5. Boundary Conditions

#### 2.5.1. Fire-Exposed Heat Boundary Conditions

(a) First-Type Boundary Condition:

The heat boundary condition at the fire-exposed surface is expressed as follows:

(22) $$q(t,0)={-h}_{s,hot}[T_{fl}(t)-T(t,0)]$$

where $h_{s,hot}$ is the convective heat transfer coefficient at the fire-exposed surface (W m^−2^ K^−1^), $T_{fl}$ is the fire temperature (K), and T(t,0) is the surface temperature at the fire-exposed side (K).

(b) Second-Type Boundary Condition:

Based on the U.S. Department of Energy’s hydrocarbon flame temperature curve, the time-dependent temperature *g*(t) is defined by the following standard curve:

(23) $$g\left(t\right)=\left\{\begin{array}{c} T_{a}+\left(T_{fl}-T_{a}-100\right)R\left(t\right),\;\;t>0\\ T_{a},\;\;t=0 \end{array}\right.$$

where $T_{a}$ is the ambient temperature on the unexposed side(K), and $R\left(t\right)$ is the temperature rise rate, defined as follows:

(24) $$R\left(t\right)=1-exp\left[-exp(0.71\;\log\;\frac{t}{124.8}\right]$$

(c) Third-type Boundary Condition:

The heat boundary conditions at the fire-exposed surface are defined as follows:

(25) $$q\left(t,0\right)=\bar{q}\left(t\right)$$

#### 2.5.2. Un-Exposed Heat Boundary Conditions

(a) First-Type Boundary Condition:

(26) $$q(t,L)=h_{s,cold}[T(t,L)-T_{a}]$$

where $h_{s,cold}$ is the convective heat transfer coefficient at the unexposed surface (W m^−2^ K^−1^).

(b) Second-Type Boundary Condition:

(27) $$q(t,L)=\bar{q}\left(t\right)$$

#### 2.5.3. Gas Mass Flux

It is assumed that decomposition gas does not escape through the backfire surface:

(28) $${\dot{m}}_{g}(t,L)=0$$

According to the boundary conditions, the gas mass flux in the system can be eliminated by integrating the following equation:

(29) $${\dot{m}}_{g}(L)-{\dot{m}}_{g}(x)=\int_{x}^{L}\frac{\partial {\dot{m}}_{g}}{\partial x}dx=-\int_{x}^{L}\frac{\partial \rho_{r}}{\partial t}dx$$

The marginal form of the formula can be derived as follows:

(30) $$r(x,t)=\rho c_{p}\frac{\partial T}{\partial t}-\frac{\partial }{\partial x}\left(k\frac{\partial T}{\partial x}\right)+\frac{\partial \rho_{r}}{\partial t}\left(h-h_{g}\right)-\left(\int_{l}^{x}\frac{\partial \rho_{r}}{\partial t}dx\right)c_{pg}\frac{\partial T}{\partial x}+Q\frac{\partial \rho_{r}}{\partial t}=0$$

It is noteworthy that the boundary condition in this model is defined such that pyrolysis gases are not permitted to escape from the unexposed surface. Consequently, gas accumulation within the material pores may lead to internal pressure buildup. However, this study does not consider the effects of internal gas-induced pressure on the mechanical properties of the composite material. Future work could extend this model to incorporate the mechanical coupling effects resulting from internal gas accumulation, enabling predictions that more closely reflect realistic conditions.

### 2.6. Solid-Phase Density Update

The update of the density term *ρ* is a critical step in the thermal response model of composite materials under fire conditions, aiming to accurately describe the material’s mass loss and structural evolution during pyrolysis. By updating *ρ*, the model ensures the closure of energy and mass conservation equations, reflecting the kinetic behavior of the decomposition reaction through the Arrhenius equation. Additionally, the use of implicit methods (e.g., the trapezoidal rule) for updating *ρ* enhances numerical stability and supports the calculation of key output variables, such as the temperature field *T*(*x*,*t*) and the gas generation rate ${\dot{m}}_{g}$. This comprehensive approach captures the thermal and structural responses of composite materials under fire exposure, providing essential theoretical support for fire safety assessment and material design. To update ρ as a fundamental variable in the formulation, its dependency on $\Theta_{m+1}$, (the updated temperature state at the next time step) is considered. Applying the trapezoidal rule, the update for *ρ* is expressed as follows:

(31) $$F_{m+1}=F_{m}+\frac{\Delta t}{2}\left({\left.\frac{\partial F}{\partial t}\right|}_{m}+{\left.\frac{\partial F}{\partial t}\right|}_{m+1}\right)$$

An update algorithm of the following form is derived:

(32) $$F_{m+1}=F_{m}+\Delta t\left.\left[-A{\left(\frac{\rho_{r}-\rho_{ch}}{\rho_{vr}-\rho_{ch}}\right)}^{n}exp\left(-\frac{E_{a}}{RT_{m}}\right)F_{m}^{n}\right.\right]$$

If n = 1, an explicit solution can be obtained:

(33) $$F_{m+1}=\frac{\frac{\Delta t}{2}F_{m}+{\left.\frac{\partial F}{\partial t}\right|}_{m}}{\frac{\Delta t}{2}+Aexp\left(-\frac{E_{a}}{RT_{m+1}}\right)}$$

In addition, the solution can be obtained directly using Newton iteration with *F*_0_=1 and the initial condition *T*_0_ = *T*_ini_.

(34) $${\left.\frac{\partial F}{\partial t}\right|}_{0}=-A{\left(\frac{\rho_{v}-\rho_{ch}}{\rho_{v}}\right)}^{n-1}exp\left(-\frac{E_{a}}{RT_{0}}\right)F_{0}^{n}$$

### 2.7. Finite Element Formulation

#### 2.7.1. Spatial Discretization

The governing equations are spatially discretized using the Galerkin finite element method with linear basis functions:

(35) $$N_{i}\left(x\right)=1-\frac{x}{l},\;N_{j}\left(x\right)=\frac{x}{l}\;(x_{i}\leq x\leq x_{j})$$

Two boundary schemes are implemented:

Scheme 1: Dirichlet conditions on both fire-exposed (*x* = 0) and unexposed (*x* = *L*) surfaces.

Scheme 2: Neumann flux at x = 0 and Dirichlet at *x* = *L*.

The weak form is derived as follows:

(36) $$\int_{0}^{L}\left.\left[N_{i}\rho c_{p}\frac{\partial T}{\partial t}+\frac{\partial N_{i}}{\partial x}k\frac{\partial T}{\partial x}\right.\right]dx+N\left(T\right)+B\left(T\right)=0\;\;\;\left(i=1,\ldots ,n_{eq}\right)$$

where $N\left(T\right)$ and $B\left(T\right)$ represent pyrolysis–gas coupling and boundary terms. The core matrices include the following:

Capacitance matrix:

(37) $$M_{ij}=\int_{0}^{L}N_{i}\rho c_{p}N_{j}dx$$

Conductivity matrix:

(38) $$K_{c,ij}=\int_{0}^{L}\frac{\partial N_{i}}{\partial x}k\frac{\partial N_{i}}{\partial x}dx$$

#### 2.7.2. Pyrolysis–Gas Coupling

Resin decomposition follows temperature-dependent Arrhenius kinetics:

(39) $$\frac{\partial \rho_{r}}{\partial t}=-A(\rho_{vr}-\rho_{ch})exp\left(-\frac{E_{a}}{RT}\right)$$

Pyrolysis-induced gas flow contributes to this convection term:

(40) $$K_{g,ij}=\int_{0}^{L}N_{i}\left(\int_{x}^{L}\frac{\partial \rho_{r}}{\partial t}dx\prime \right)c_{pg}\frac{\partial N_{i}}{\partial x}dx$$

For Scheme 2, the fire-side heat flux dynamically updates using previous-step temperatures:

(41) $$q_{fire}^{m+1}=h_{hot}{(T}_{f1}-T_{1}^{m})$$

#### 2.7.3. Matrix Optimization

Leveraging uniform 1D meshing (Δ*x* = *l*), analytical integration yields the following:

(42) $$M=\frac{\rho c_{p}l}{6}\left[\begin{array}{cc} 2 & 1\\ 1 & 2 \end{array}\right],\;\;\;K_{c}=\frac{k}{l}\left[\begin{array}{cc} 1 & -1\\ -1 & 1 \end{array}\right]$$

### 2.8. Temporal Discretization

A partitioned scheme enhances stability and efficiency.

(a) Implicit Crank–Nicolson for Diffusion:

(43) $$\left(\frac{\Delta t}{2}M+\tilde{K}\right)T_{m+1}=\frac{\Delta t}{2}MT_{m}+L_{m+1},\;\;\;\tilde{K}=K_{c}+K_{d}+K_{g}+K_{s}$$

(b) Explicit Update for Pyrolysis:

(44) $$T_{m+1}={\left(M+\frac{\Delta t}{2}\tilde{K}\right)}^{-1}\left(M-\frac{\Delta t}{2}\tilde{K}\right)T_{m}-\frac{\Delta t}{2}L_{m}$$

### 2.9. The Finite Element Program

A computational framework was developed for simulating combustion-induced heat transfer in composite materials, integrating finite element analysis with thermodynamic principles. The Python version 2.7 based implementation utilizes PyCharm IDE and incorporates scientific libraries, including pandas for data structuring, numpy for matrix operations, matplotlib for graphical outputs, and PyQt5 for GUI construction. The system architecture employs two functionally independent modules: a GUI frontend managing parameter configuration and visualization tasks, and a computational backend executing finite element simulations. Inter-module communication is achieved through Python dictionary objects, where the GUI serializes user inputs (material properties, boundary conditions, and numerical schemes) into key–value pairs, while the computation module returns JSON-formatted results containing temperature field and reaction kinetic data. This decoupled design ensures computational scalability, while maintaining interactive visualization capabilities through dynamic plotting functions.

### 2.10. Parameter Input

The finite element program’s input interface encompasses the material properties, the decomposition reaction parameters, the finite element solution parameters, and the computational boundary and initial conditions. The material properties section requires an input for the thermal conductivity *k* (W·m^−1^·K^−1^), specific heat capacity c_p_ (J·kg^−1^·K^−1^), and density *ρ* (kg·m^−3^) of the composite material *ρ*_c_, matrix, the reinforcement fibers *ρ*_matrix_, and the carbon layer *ρ*_fiber_, as well as the specific heat capacity of the decomposition gases c_p,g_. The decomposition reaction parameters include the decomposition reaction rate (pre-exponential factor A (s^−1^), activation energy *E*a (kJ·kmol^−1^), the gas constant R (kJ·kmol^−1^·K^−1^), and reaction order n), the fiber volume fraction *V*_f_, and the heat of decomposition Δ*H* (J·kg^−1^). The finite element solution parameters involve inputs such as plate thickness (m), the number of nodes *N*, the time step size Δ*t*(s), total simulation time *t*_tot_(s), residual convergence criteria *ϵ*, and the selection of numerical methods for spatial and temporal solutions, including spatial discretization schemes (e.g., 1/2) and implicit/explicit time schemes. The computational boundary and initial conditions section requires the flame temperature *T*_flame_ (K), the ambient temperature *T***_∞_** (K), the heat transfer coefficients *h* (W·m^−2^·K^−1^) on the exposed and unexposed surfaces, and heat flux density *q* (W·m^−2^) on both surfaces. These inputs collectively enable the precise and comprehensive simulation of the thermo-physical behavior within the finite element model. The typical data input parameters for composite materials are referenced in [45], as shown in Table 1.

## 3. Data Validation

**Figure 1:**
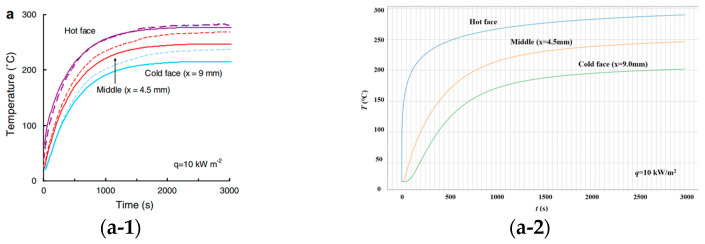

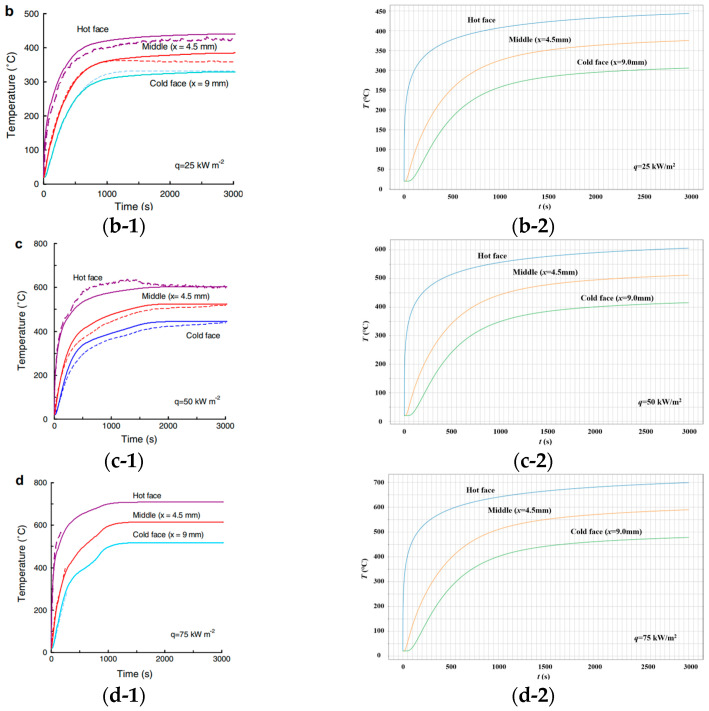
Temperature rise curve comparison. (**a-1**) *q* = 10 kW/m^2^ [45]. (**a-2**) *q* = 10 kW/m^2^. (**b-1**) *q* = 25 kW/m^2^ [45]. (**b-2**) *q* = 25 kW/m^2^. (**c-1**) *q* = 50 kW/m^2^ [45]. (**c-2**) *q* = 50 kW/m^2^. (**d-1**) *q* = 75 kW/m^2^ [45]. (**d-2**) *q* = 75 kW/m^2^.

Figure 1 presents the temperature rise curves at the hot face, the mid-thickness section, and the cold face of the material under the heat flux conditions of 10 kW/m^2^, 25 kW/m^2^, 50 kW/m^2^, and 75 kW/m^2^. Under different heat flux conditions, the temperature rise trends in this study are generally consistent with those reported in the literature. However, a notable difference is observed in the initial heating rate at the hot face. Specifically, the hot face in this study exhibits a higher temperature rise rate and reaches steady state more quickly across all the heat flux levels. This discrepancy is primarily attributed to the differences in the system boundary conditions, such as the heating method applied to the hot surface, the thermal contact conditions, and the surface radiation characteristics, which result in a more rapid thermal response. In the steady-state phase, both the hot and cold face temperatures in this study are slightly lower than those in the literature, which may be related to the better thermal insulation of the specimen or the more effective control of heat losses in the experimental system.

**Figure 2:**
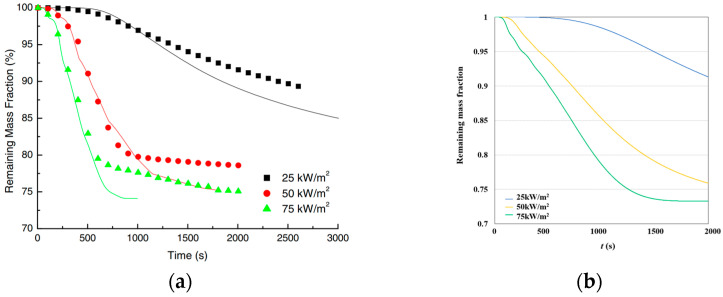
Remaining mass fraction curve comparison. (**a**) Literature data. (**b**) Calculated data.

As shown in Figure 2, both this study and the literature show a consistent trend where the remaining mass fraction decreases over time, with higher heat flux levels accelerating the degradation process.

**Figure 3:**
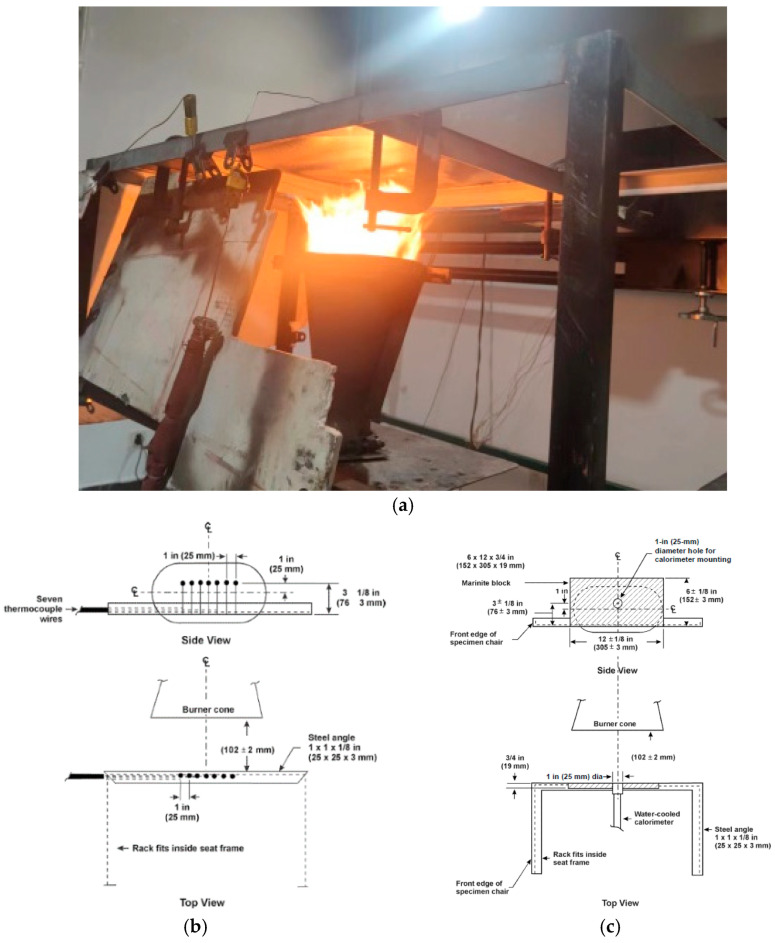
Experimental photograph and schematic diagram of calibration method. (**a**) Experimental photograph. (**b**) Temperature calibration [46]. (**c**) Heat flux calibration [46].

The finite element program was validated against experimental tests conducted in accordance with the 14 CFR Part 25 [46] and ISO 2685 [47] standards. As shown in Figure 3, the burner and flame calibration setup included a thermocouple rake with seven thermocouples positioned 102 ± 2 mm from the burner outlet plane, ensuring the alignment of the thermocouple tips with the burner centerline. A heat flux meter was installed at the endpoint of the fourth thermocouple on the rake.

**Figure 4:**
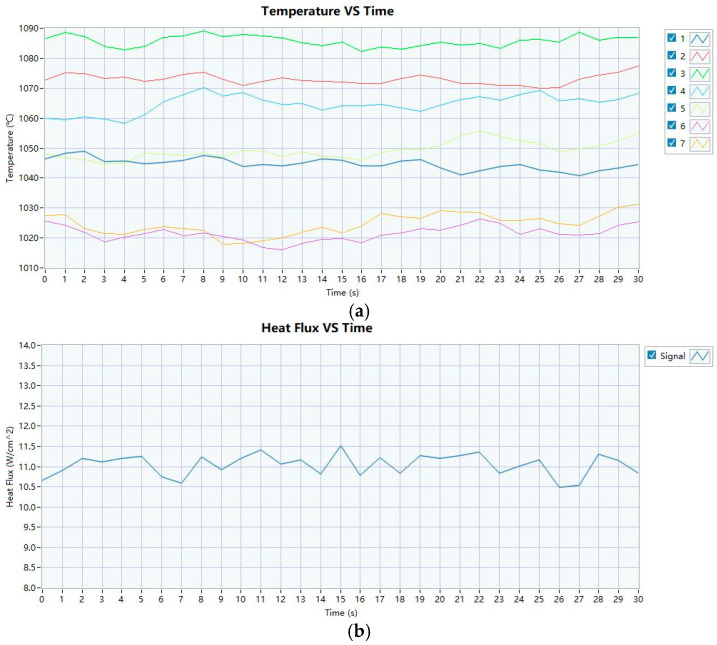
Temperature and heat flow calibration data. (**a**) Temperature calibration data. (**b**) Heat flux calibration data.

During system calibration, the relative positions between the thermocouples, the heat flux meter, and the burner were rigorously verified to ensure geometric consistency. For flame characterization, the thermocouple rake was fixed on the test platform, while the burner was translated to its preheating position. Temperature profiles from the seven thermocouples were recorded over a 30 s interval at 1 Hz sampling frequency, yielding an averaged temperature of 1100 ± 80 °C. Following thermal stabilization, the rake was repositioned to the preheating location, concurrently aligning the heat flux meter at the measurement station to confirm compliance with the minimum 10.6 W/cm^2^ flux threshold. Iterative burner adjustments were performed until both the temperature and heat flux criteria were satisfied. The specimen was mounted on a dedicated holder, ensuring full flame coverage over a 127 mm × 127 mm (5 in × 5 in) surface area. Upon burner ignition and preheating, the flame source was rapidly positioned at the test location to initiate timing. Throughout the 5 min fire exposure, the time-resolved temperature and heat flux data were acquired from the flame-impinged surface. The calibration metrics for the thermal parameters are summarized in Figure 4.

**Figure 5:**
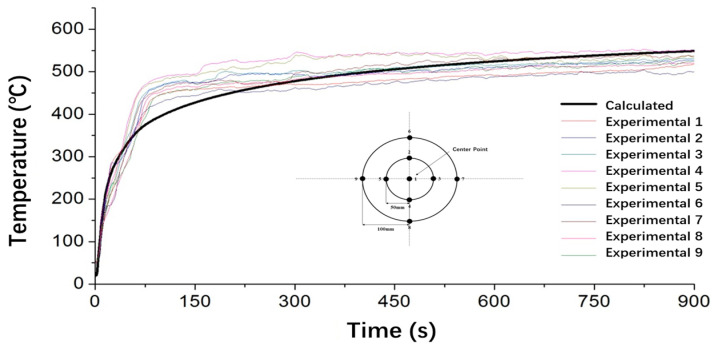
Comparison of predicted and experimental values.

Figure 5 presents a comparison between the experimentally measured temperature distribution on the unexposed surface of the specimen and the corresponding finite element predictions. The measured and simulated results exhibit good quantitative agreement, with relative errors consistently maintained within a small range throughout the 5 min exposure period, thereby validating the computational accuracy of the model. This indicates that the developed model is capable of effectively resolving the coupled heat transfer mechanisms occurring during combustion, including thermal conduction through the composite layers, convective heat exchange at the boundary layer, and energy absorption associated with pyrolysis. However, as shown in the figure, the calculated curve exhibits limited sensitivity to short-term temperature fluctuations, failing to capture the transient variations observed in the experiments. This highlights the need for the further refinement of the boundary condition settings to better reflect actual conditions and improve the model’s dynamic response and overall predictive accuracy.

## 4. Conclusions

This study develops a comprehensive mathematical model to predict the thermal response, decomposition behavior, and gas generation dynamics of composite materials under high-temperature conditions. The model dynamically incorporates the variations in thermophysical properties—such as thermal conductivity, specific heat capacity, and density—using the Rule of Mixtures. By employing the Arrhenius equation to characterize matrix decomposition kinetics and its coupling effects with heat transfer, the framework addresses the limitations in prior research related to simplified gas generation processes and static property assumptions. Experimental validation conducted in accordance with the 14 CFR Part 25 and ISO 2685 standards confirms the model’s predictive accuracy. The model successfully resolves critical phenomena observed in other experiments, including the char layer insulation effects, transient heat flux attenuation due to gas convection, and the decomposition-induced variations in material properties, thereby highlighting the necessity of integrating gas generation dynamics into energy conservation equations. This computational tool enables the precise evaluation of composite fire resistance, providing a basis for optimizing material parameters (e.g., the fiber volume fraction, the pre-exponential factor, and activation energy) and boundary conditions (heat flux and convective coefficients). However, the current 1D formulation assumes instantaneous gas dispersion and uniform property transitions, which may underestimate the 3D thermal–mechanical coupling effects. Future extensions will incorporate multidimensional heat transfer and hygroscopic behavior to enhance real-world applicability, advancing the fire safety design methodologies for aerospace structures.

## Figures and Tables

**Table 1:** Material property parameters [45].

Property	Value
Pre-exponential factor, A (1/s)	5.59 × 10^13^
Specific heat (fiber), (J/(kg K))	760.0
Specific heat (resin), (J/(kg K))	1600.0
Specific heat (char) (J/(kg K))	1289
Specific heat (gas), (J/(kg K))	2386.5
Activation energy, (J/mol)	50,000
Thermal conductivity (fiber), (W/(m K))	1.04
Thermal conductivity (resin), (W/(m K))	0.20
Thermal conductivity (char), (W/(m K))	1.2
Heat of decomposition, (J/kg)	2.3446 × 10^5^
Ambient temperature, (°C)	20.0
Volume fraction	0.55
Density (initial), (kg/m^3^)	1812.0
Density (fiber), (kg/m^3^)	2560.0
Density (resin), (kg/m^3^)	1200.0
Density (vinyl ester), (kg/m^3^)	1300
Order of decomposition reaction	1

## Data Availability

The original contributions presented in this study are included in the article. Further inquiries can be directed to the corresponding author.
